# Cigarette Smoke Specifically Affects Small Airway Epithelial Cell Populations and Triggers the Expansion of Inflammatory and Squamous Differentiation Associated Basal Cells

**DOI:** 10.3390/ijms22147646

**Published:** 2021-07-16

**Authors:** Christian T. Wohnhaas, Julia A. Gindele, Tobias Kiechle, Yang Shen, Germán G. Leparc, Birgit Stierstorfer, Heiko Stahl, Florian Gantner, Coralie Viollet, Jürgen Schymeinsky, Patrick Baum

**Affiliations:** 1 Boehringer Ingelheim Pharma GmbH & Co. KG, 88397 Biberach, Germany; julia_anna.gindele@boehringer-ingelheim.com (J.A.G.); tobias.kiechle@boehringer-ingelheim.com (T.K.); yang_3.shen@boehringer-ingelheim.com (Y.S.); german.leparc@boehringer-ingelheim.com (G.G.L.); birgit.stierstorfer@boehringer-ingelheim.com (B.S.); heiko.stahl@boehringer-ingelheim.com (H.S.); florian.gantner@boehringer-ingelheim.com (F.G.); coralie.viollet@boehringer-ingelheim.com (C.V.); juergen.schymeinsky@boehringer-ingelheim.com (J.S.); patrick.baum@boehringer-ingelheim.com (P.B.); 2 Department of Biology, University of Konstanz, 78457 Konstanz, Germany

**Keywords:** single-cell transcriptomics, small airway epithelial cells, cigarette smoke, COPD, basal cells, lineage trajectory, ACE2

## Abstract

Smoking is a major risk factor for chronic obstructive pulmonary disease (COPD) and causes remodeling of the small airways. However, the exact smoke-induced effects on the different types of small airway epithelial cells (SAECs) are poorly understood. Here, using air–liquid interface (ALI) cultures, single-cell RNA-sequencing reveals previously unrecognized transcriptional heterogeneity within the small airway epithelium and cell type-specific effects upon acute and chronic cigarette smoke exposure. Smoke triggers detoxification and inflammatory responses and aberrantly activates and alters basal cell differentiation. This results in an increase of inflammatory basal-to-secretory cell intermediates and, particularly after chronic smoke exposure, a massive expansion of a rare inflammatory and squamous metaplasia associated *KRT6A*^+^ basal cell state and an altered secretory cell landscape. ALI cultures originating from healthy non-smokers and COPD smokers show similar responses to cigarette smoke exposure, although an increased pro-inflammatory profile is conserved in the latter. Taken together, the in vitro models provide high-resolution insights into the smoke-induced remodeling of the small airways resembling the pathological processes in COPD airways. The data may also help to better understand other lung diseases including COVID-19, as the data reflect the smoke-dependent variable induction of SARS-CoV-2 entry factors across SAEC populations.

## 1. Introduction

Airway epithelial cells are a community of diverse cell types that constitute a physical barrier to the environment and contribute to homeostasis in the airways by regulating immune responses, mucociliary clearance and response to inhaled toxins [1,2,3]. These functions are managed by an interplay of the different epithelial cell types including basal, ciliated and secretory (club and goblet) cells, which constitute the main cell types as well as rare epithelial cells such as pulmonary ionocytes [1]. Among these cell types, basal cells act as important progenitors that give rise to secretory and ciliated cells and play a critical role in homeostatic maintenance and repair of the airway epithelium [4,5,6,7,8]. The role of intermediate cell states is less well understood. Dysfunction of the dynamic airway epithelium, including cellular alterations and altered molecular signaling, in response to inhaled toxins such as cigarette smoke (CS) has been linked to lung diseases.

Smoking is the most important risk factor of developing chronic obstructive pulmonary disease (COPD) [9,10]. The small airways are the major site of airflow limitation in COPD [11,12] but the exact effect of CS exposure on the various small airway epithelial cell (SAEC) populations remains incompletely understood. Previous studies revealed aberrations of the small airway epithelium in COPD patients and smokers, such as goblet cell metaplasia [12,13,14], and suggested that basal cells are the first cell type affected by CS [15,16,17,18]. Injured basal cells are impaired in their differentiation capacity, which leads to remodeling of the airways that is characteristic for the COPD histopathology [15,19,20,21], including basal cell hyperplasia, squamous metaplasia [22,23,24,25] and reduced cilia function [26,27,28,29]. Transcriptomic analyses of smokers further demonstrated substantial effects of CS exposure on the small airway epithelium [30,31]. However, these transcriptomic studies were performed on the bulk level and did not allow investigation of the smoke effect on individual SAEC populations. Single-cell RNA-sequencing (scRNA-seq) allows dissection of the CS-induced transcriptomic effects on the cellular level but recent studies mainly focused on the large airways [3,32,33]. Only little is known about the specific molecular responses of SAEC populations, their molecular phenotypes, and their role in airway remodeling.

Here, acute and chronic CS-induced effects on the SAEC landscape were investigated in a controlled environment using scRNA-seq and primary cell derived SAEC air–liquid interface (ALI) cultures originating from healthy controls (HC) and COPD patients. This study provides new insights into the transcriptional SAEC landscape at the single-cell level and dissects smoke-induced, cell-type specific inflammatory and detoxification responses. Furthermore, it shows cell-type specific CS-dependent induction of SARS-CoV-2 entry factors. This work also characterizes a subset of inflammatory, squamous metaplasia associated basal cells whose molecular profile is related to pathologically relevant molecular processes seen in COPD.

## 2. Results

### 2.1. Small Airway Epithelial Cells—A Heterogeneous Cell Population

**Figure 1:**
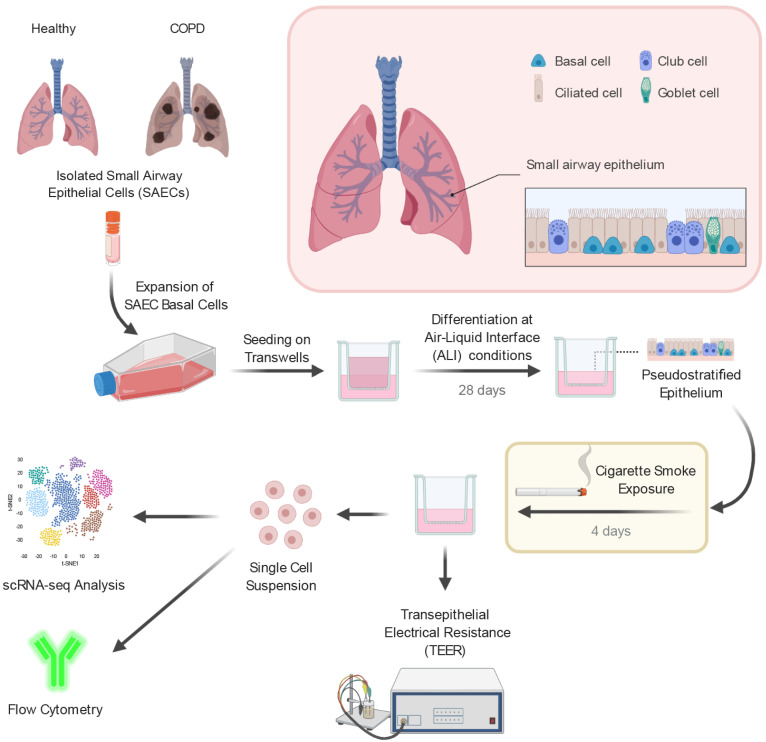
Experimental design to investigate the impact of acute smoke exposure on small airway epithelial cells. Basal cells from primary small airway epithelial cells (SAECs) isolated from lungs of healthy controls (*n* = 3) and chronic obstructive pulmonary disease (COPD) patients (*n* = 3) were expanded in vitro and differentiated on air–liquid interface (ALI) conditions into a pseudostratified epithelium. Characterization and investigation of acute cigarette smoke exposure (4 days) on the different SAEC populations were performed by single-cell RNA-sequencing (scRNA-seq), flow cytometry and transepithelial electrical resistance measurement.

In order to investigate the cellular composition of the small airway epithelium, isolated small airway epithelial cells from healthy non-smokers and COPD donors (*n* = 3 per group) were differentiated under air–liquid interface conditions into a pseudostratified epithelium [34]. To mimic smoke-induced injuries to the small airways of healthy non-smokers and COPD smokers, the fully differentiated SAEC ALI cultures were exposed either to whole CS for four consecutive days or to ambient air as control (Figure 1). Subsequently, the ALI cultures were analyzed by means of flow cytometry, transepithelial electrical resistance (TEER) measurement and scRNA-seq (Figure 1).

**Figure 2:**
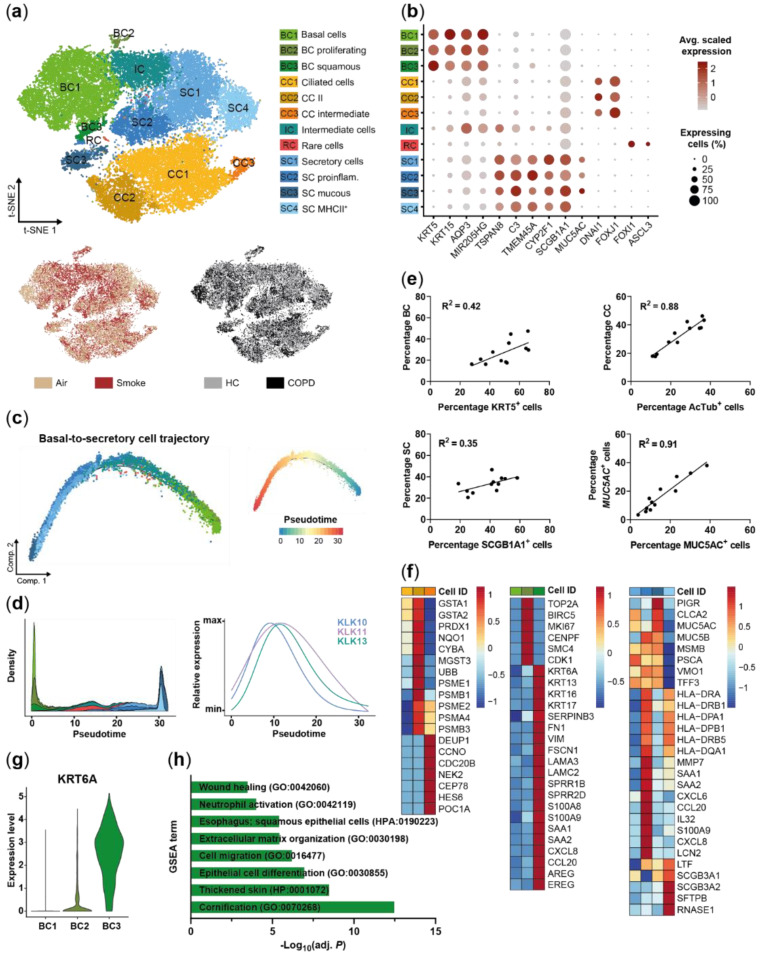
Characterization of the small airway epithelial cell population. (**a**) T-SNE plot illustrating the 12 clusters identified in the integrated scRNA-seq data sets of all donors (*n* = 3 healthy controls (HC) and COPD patients, respectively) including three basal cell (BC) clusters, three ciliated cell (CC) clusters, a basal/secretory intermediate cell (IC) cluster, a rare cell (RC) cluster and four secretory cell (SC) clusters. T-SNE plots in the lower panel illustrate cells by smoking and disease status. (**b**) Relative expression and fraction of cells that express BC (*KRT5*, *KRT15*), CC (*FOXJ1*, *DNAI1*), SC (*CYP2F1*, *SCGB1A1*) and RC (*FOXI1*, *ASCL3*) marker genes across the cell clusters. Low expression levels of typical BC and SC markers are detected in IC, however, IC express a set of genes that are strongly expressed in BC or SC (*AQP3*, *MIR205HG*, *TSPAN8*, *C3*). (**c**) Pseudotime analysis of SAEC populations (*n* = 6, air control and smoke-exposed samples of HC) focused on BC/IC/SC relationship. Trajectories show annotated cell IDs (left) using the color code from (**a**) and pseudotime values (right) per cell. (**d**) Left panel represents density (stacked) of the SAEC populations along pseudotime. The right panel depicts the modelled, relative expression of selected kallikrein-related peptidase encoding genes along pseudotime. (**e**) Correlation of relative cell type frequencies (HC and COPD donors) as determined by scRNA-seq (y-axis) and established protein markers of the respective cell types using flow cytometry (x-axis). (**f**) Heatmaps depict relative expression levels of selected genes that are significantly differentially expressed (adj. *p* < 0.05, abs. fold change ≥1.5) across CC, BC and SC subpopulations. (**g**) Normalized *KRT6A* expression levels in cells of the three BC subpopulations. (**h**) Selected molecular processes that are significantly (adj. *p* < 0.05) associated with BC3 as determined by gene set enrichment analysis (GSEA) of the genes significantly increased compared to BC1 and BC2. AcTub: acetylated-tubulin.

A single cell suspension was generated of the differently treated ALI cultures and a total of 34,690 cells were sequenced to investigate cell-specific heterogeneity and response to smoke on the transcriptomic level. Unsupervised cluster analysis of all detected transcripts identified 12 distinct clusters in the SAEC population (Figure 2a). Cells did not cluster by treatment (smoke vs. air control) or donor group (HC vs. COPD), indicating no underlying experimental bias (Figure 2a). Clusters were also not driven by donor variability as cells from each donor were spread across all clusters (Figure S1a).

Selective expression of known marker genes revealed three basal cell clusters (*KRT5*^+^, *KRT15*^+^), three ciliated cell clusters (*FOXJ1*^+^, *DNAI1*^+^) and four secretory cell clusters (*CYP2F1*^+^, *SCGB1A1*^Hi^) in the SAEC population. Cells expressing the goblet cell marker *MUC5AC* were enriched within the secretory cell population but did not form a separate cluster (Figure 2b and Figure S1b). Further analysis revealed a small cluster of 63 cells (0.2% of all cells) that represented rare cell types including *FOXI1*^+^ pulmonary ionocytes and tuft-like cells (Figure S1c).

In addition to the basal and secretory cell clusters, a distinct intermediate cell cluster was identified. This intermediate cluster did not express the typical cell type markers, but instead expressed genes found in either basal or secretory cells. For example, *AQP3* and the lincRNA *MIR205HG* showed a strong expression in basal and intermediate cells and lower expression in secretory cells. In contrast, *TSPAN8* and *C3* were both expressed by secretory and intermediate cells but were absent or lower expressed in basal cells. However, two additional secretory cell markers *TMEM45A* and *CYP2F1* were weakly expressed or absent in intermediate and basal cells (Figure 2b). Finally, trajectory analysis was performed to further investigate the basal-to-secretory cell transition based on the gene expression profiles of these cell populations. The intermediate cells were positioned in the middle of the differentiation trajectory confirming their differentiation status between basal cells and secretory cells, i.e., intermediate cellular state by an alternative method (Figure 2c). Several members of the kallikrein-related peptidase (KLK) serine protease family (*KLK10*, *KLK11*, *KLK13*) showed a significantly differential expression along the differentiation trajectory with a peak expression around maximum intermediate cell density, suggesting a novel role of these KLK members in basal-to-secretory cell differentiation (Figure 2d).

In parallel to scRNA-seq the frequency of the main SAEC cell types KRT5^+^ basal cells, acetylated tubulin positive ciliated cells, SCGB1A1^+^ secretory club cells and MUC5AC^+^ secretory goblet cells was determined by flow cytometry. The relative frequencies determined by flow cytometry and scRNA-seq showed a moderate correlation for basal (R^2^ = 0.42) and secretory cells (R^2^ = 0.35). In contrast, a strong correlation was observed for ciliated and MUC5AC^+^ cells (R^2^ ≥ 0.88), which demonstrates that the relative cellular SAEC composition is reflected in the scRNA-seq data (Figure 2e).

Next, the different subpopulations identified by cluster analysis were investigated (Figure 2a). In-depth analysis of these subpopulations revealed that they represent different cell states or distinct cell subtypes. Ciliated cells could mainly be separated into intermediate multiciliated cells (CC3) that showed a characteristic expression of deuterosomal and centriole formation associated genes (*DEUP1, CCNO, CDC20B, NEK2, CEP78, PCM1, POC1A*) and mature ciliated cells (CC1 and CC2, Figure 2a,f). The mature ciliated cell clusters were distinguished by a differential expression of oxidative stress, detoxification and proteasome associated genes (*GSTA1, GSTA2, NQO1, CYBA, PSMB1, PSME2*) (Figure 2f). Basal cells were subdivided into three subpopulations including a cluster (BC2) that specifically expressed proliferation associated genes (*TOP2A, BIRC5, MKI67, CENPF, SMC4, CDK1*) and therefore identified as proliferating basal cells (Figure 2a,f). A small basal cell cluster (BC3) representing 5.3% of the basal cells was instead characterized by strong expression of *KRT6A*, which is associated with squamous differentiation [35,36] (Figure 2g). Differential gene expression and gene set enrichment analysis furthermore identified an upregulation of genes, processes and pathways related to cell differentiation and cell migration (*KRT6A*, *KRT16*, *KRT17*, *SERPINB3*, *FN1, VIM, FSCN1, LAMA3, LAMC2, SPRR1B, SPRR2D*) including the barrier alarmins *KRT16/17* [37] and the EGF receptor ligands *AREG* and *EREG* (Figure 2f,h). Moreover, this basal cell cluster showed an increased expression of pro-inflammatory mediators (*S100A8/9, SAA1, SAA2, CXCL8, CCL20*) indicating an activated state of these basal cells (Figure 2f). Secretory cells were the most heterogeneous cell population and distinct transcriptional signatures of the four cell clusters indicate functional diversity within the secretory cells. Known secretory cell markers [1,38] showed a differential expression across the subpopulations with an increased expression of the secretoglobins *SCGB3A1* and *SCGB3A2* in secretory cell cluster 4 (SC4), whereas *MSMB, TFF3, MUC5B* and *PIGR* were only weakly expressed by this subpopulation (Figure 2f). In contrast, SC3 cells showed a strong *PIGR* and mucin (*MUC5B*, *MUC5AC*) expression, which is in line with their localization at the end of the basal-to-secretory cell differentiation trajectory and potentially more mature secretory cell state (Figure 2c,f). In addition to the heterogeneity of known secretory cell markers, a variable expression of MHC-II encoding genes was detected across the clusters. A set of MHC-II chain encoding genes (*HLA-DRA, HLA-DRB1*, *HLA-DPA1*, *HLA-DPB1*, *HLA-DRB5*, *HLA-DQA1*) was strongly expressed by SC2 and SC4 but not by SC1 and SC3. The remaining marker genes of the two MHC-II^Hi^ subpopulations suggest a different role of these subpopulations (Figure 2f). SC2 was associated with a pro-inflammatory response as exemplified by anup-regulated expression of antimicrobial mediators, interleukins and chemokines (SAA1, SAA2, *CXCL6, CCL20, S100A9, CXCL8, LCN2)*. In contrast, SC4 selectively expressed *RNASE1* as well as *SFTPB* and *SCGB3A2*, which are linked to anti-inflammatory processes [39,40,41,42]. SC4 was also characterized by an overall decreased expression of mucins and mucus-associated genes (*MUC5B*, *TFF3*, *PSCA*, *MUC5AC*, *CLCA2*) [43], supporting functional heterogeneity within the secretory cell population (Figure 2f).

Taken together, a complex SAEC architecture was identified that is characterized by functionally distinct cell subpopulations and differentiation states including a basal-to-secretory intermediate state. Secretory cells represent the most heterogeneous population, whereas a small pro-inflammatory and squamous differentiation associated *KRT6A*^+^ cell cluster was identified in basal cells.

### 2.2. Disease-Relevant Characteristics in COPD-Derived ALI Cultures and Their Differences in Response to Cigarette Smoke Exposure

Beyond investigating the complexity of the human SAEC population, it was investigated whether COPD-relevant characteristics are conserved in ALI cultures and whether COPD-derived cultures respond differently to CS compared to HC. All cell subpopulations identified by cluster analysis were detected in HC- and COPD-derived ALI cultures, indicating a similar cellular profile of SAEC ALI cultures from both groups (Figure 2b and Figure S1d).

**Figure 3:**
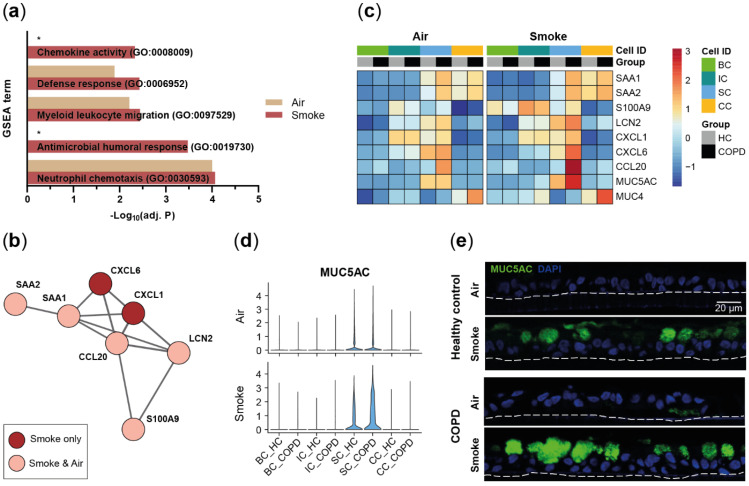
COPD-derived SAECs are characterized by a pro-inflammatory gene expression profile. (**a**) GSEA on the genes up-regulated in COPD compared to HC in at least one cell type. Selected significantly (adj. *p* < 0.05) enriched molecular processes are shown for air control and smoke-exposed cells. (**b**) Protein–protein interaction network of pro-inflammatory and anti-microbial genes that are increased in air control and/or smoke-exposed cells of COPD donors. (**c**) Relative expression of the gene set from (**b**) and de-regulated mucin encoding genes across the main SAEC populations from HC- and COPD-derived air control and smoke-exposed ALI cultures. (**d**) Violin plots illustrate *MUC5AC* expression across the main cell types in air control and smoke-exposed SAECs from COPD and HC donors. (**e**) Representative immunofluorescence labelling of MUC5AC in air control and smoke-exposed SAEC sections from HC and COPD donors. *: pathway not significantly enriched (adj. *p* > 0.05).

To elucidate potential transcriptional differences between COPD- and HC-derived SAEC populations, differential gene expression analysis was performed on the main SAEC populations (basal, intermediate, secretory and ciliated cells). Air and smoke-exposed ALI cultures from both donor groups showed 25 and 36 differentially expressed genes in at least one main SAEC population, respectively. Functional enrichment of biological processes associated with immune response and defense mechanisms was observed for the genes increased in COPD donors (Figure 3a). Similar processes were observed in air- and smoke-exposed cultures although the effect was stronger in smoke-exposed cells (Figure 3a–c). The gene set of de-regulated pro-inflammatory mediators included acute phase and antimicrobial markers (*SAA1, SAA2, S100A9, LCN2*) and chemokines related to neutrophil (*CXCL1*, *CXCL6*) [44,45] and dendritic cell/lymphocyte (*CCL20*) recruitment [46,47,48].

An increased expression of these pro-inflammatory markers was observed in ciliated and/or secretory cells of COPD ALI cultures. Most of the genes were differentially expressed in smoke-exposed and air control ALI cultures with exception of CXC*L1* and *CXCL6* were only differentially expressed in secretory cells of smoke-exposed ALI cultures (Figure 3b,c). Similarly, smoke-induced expression of the goblet cell marker *MUC5AC* was stronger in the secretory cells of the COPD donors than HC; whereas, *MUC4* was increased in smoke-exposed ciliated cells of the COPD donors compared to HC (Figure 3c,d). In line with the gene expression data, semi-quantitative immunohistochemistry corroborated the elevated MUC5AC expression upon smoke exposure particularly in COPD-derived ALI cultures (Figure 3e).

Taken together, HC- and COPD-derived SAECs show similar gene expression profiles although the ALI cultures originating from COPD donors were characterized by a more inflammatory gene expression profile.

### 2.3. Acute Smoke Exposure Triggers Common and Cell Type-Specific Detoxification and Inflammatory Responses and Aberrant Basal Cell Activation and Differentiation

**Figure 4:**
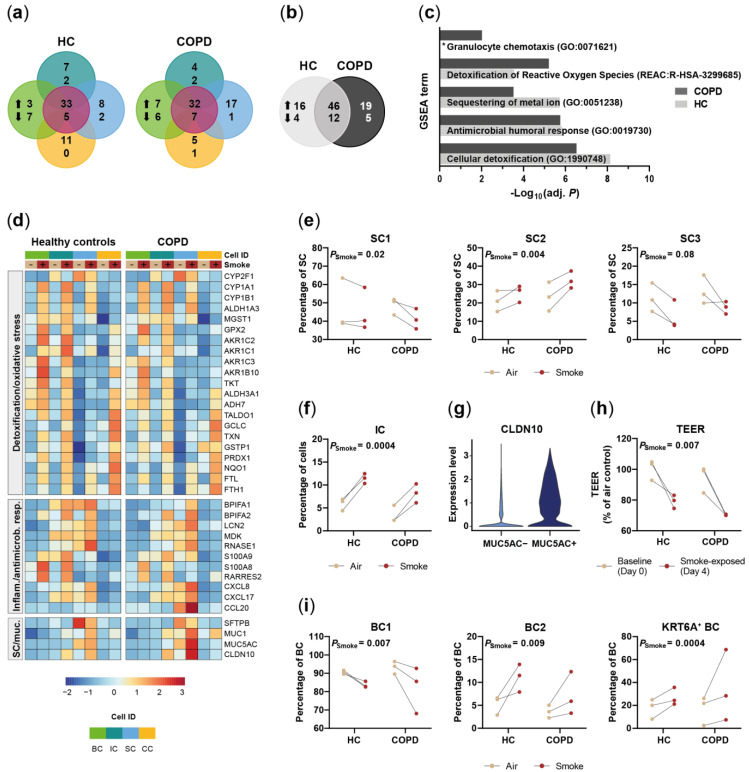
Acute smoke exposure distinctly alters gene expression and frequency of SAEC populations. (**a**) Summary of the significantly up- and down-regulated genes (adj. *p* < 0.05, abs. fold change ≥1.5) by acute smoke exposure across the main SAEC populations (BC, IC, SC and CC according to the color code in (**d**)) of HC and COPD donors (*n* = 3, each). Core genes de-regulated in at least two cell types are summarized in the center (violet circle). (**b**) Comparison of the genes that are significantly de-regulated by acute smoke exposure in at least one of the four main cell populations of HC and COPD donors. (**c**) GSEA on the up-regulated genes from (**b**). Significantly (adj. *p* < 0.05) enriched molecular processes are shown for HC and COPD. (**d**) Relative expression of genes associated with cellular detoxification/oxidative stress, inflammatory/antimicrobial response and mucus production/secretory cells across the four main SAEC populations. Average gene expression is shown for air control and smoke-exposed cells from both, HC and COPD donors. (**e**,**f**,**i**) Smoke-induced shifts of selected SC and BC subpopulation frequencies and the total IC frequency are depicted in dot plots. Data are linked by donor. (**g**) Violin plot illustrating *CLDN10* expression levels in *MUC5AC*^+^ and *MUC5AC*^-^ SC cells. (**h**) Shift in transepithelial electrical resistance (TEER) per HC and COPD donor after acute smoke exposure (Day 4) compared to baseline (Day 0). *: pathway not significantly enriched (adj. *p* > 0.05).

To assess the effect of acute smoke exposure on the small airway epithelium, we investigated whether smoke alters the frequency and gene expression profiles of the different cell (sub)populations. Differential gene expression analysis identified 78 and 82 genes that were differentially expressed in at least one of the four main cell types by smoke exposure in HC- and COPD-derived ALI cultures, respectively (Figure 4a,b). Most of the genes were induced, whereas only few genes were downregulated by CS exposure.

Around half of the up-regulated genes were induced in at least one cell type while the remaining genes showed a cell-type specific induction, indicating shared and unique responses across the main SAEC populations (Figure 4a). These smoke-induced genes showed a strong overlap between COPD- and HC-derived ALI cultures, which demonstrates a similar response for both donor groups (Figure 4b). Consequently, gene set enrichment analysis (GSEA) revealed an induction of antimicrobial, pro-inflammatory and cellular detoxification related processes by acute smoke exposure in both, HC- and COPD-derived ALI cultures (Figure 4c).

Increased oxidative stress and detoxification responses were observed after smoke exposure regardless of the cell types but the respective gene sets were different across the cell types (Figure 4d). The cytochrome P450 encoding genes *CYP1A1* and *CYP1B1*, for example, showed more than a 3-fold increase in smoke-exposed basal, intermediate and secretory cells; whereas, either less than 2-fold induction or no induction was observed in ciliated cells. In contrast, *CYP2F1* was significantly decreased only in smoke-exposed intermediate and secretory cells (Figure 4d). Cellular detoxification and oxidative stress response associated genes (*TXN*, *PRDX1*, *GPX2*, *GSTP1*, *GCLC*, *TKT*, *NQO1*, *MGST1, FTL, FTH*, aldo-keto reductase and dehydrogenase encoding genes) also showed cell type-specific induction patterns and the overall highest expression levels in basal and intermediate cells (Figure 4d). Iron homeostasis and oxidative stress associated ferritin subunits were induced in all cell types [49]. Differences in cell type-specific responses were even more pronounced for host defense and antimicrobial markers (*BPIFA1, BPIFA2, LCN2, MDK, RNASE1, S100A8/9*) and pro-inflammatory chemokines that are involved in the recruitment of a variety of immune cells including neutrophils, macrophages, dendritic cells and lymphocytes (*RARRES2*, *CXCL8*, *CXCL17*, *CCL20*) (Figure 4d). These genes showed low basal levels in ciliated cells and were only weakly induced or not induced at all in ciliated cells upon smoke exposure (Figure 4d). In contrast, most genes were induced in secretory cells and were most strongly expressed in secretory and intermediate cells, which indicates a more prominent role of these cell types in host defense and immune cell recruitment (Figure 4d). This is in line with a significant increase of pro-inflammatory secretory cells (SC2), whereas the frequency of the non-activated secretory cell cluster (SC1) decreased after acute smoke exposure (Figure 4e). We also observed a trend (*p* = 0.08) towards a decrease of the SC3 cluster that according to pseudotime analysis represented a terminally differentiated *MUC5B* expressing secretory cell state (Figure 2c,f and Figure 4e). Paradoxically, inflammatory response associated intermediate cells increased strongly in smoked ALI cultures, suggesting an aberrant basal-to-secretory cell differentiation (Figure 4f).

Secretory cells also showed an altered secretory profile that was characterized by an increased expression of *MUC1* and *MUC5AC*, although the latter was only significantly induced in COPD donors, and a down-regulation of SC4 associated *SFTPB* (Figure 2f and Figure 4d). The *MUC5AC*^+^ secretory cells co-expressed *CLDN10,* which encodes the pore-forming junctional protein claudin-10 [50,51] that might be related to the impaired barrier integrity of the small airway epithelium as determined by a significantly decreased TEER after acute smoke exposure (Figure 4h). Expression of the early barrier alarmins KRT16, KRT17 and KRT6 by keratinocytes is associated with barrier breach and hyperproliferation, suggesting that the expression of these markers in the BC3 cluster is associated with impaired barrier integrity of the small airway epithelium [37]. In agreement with that, we observed a significantly increased frequency of BC expressing the squamous differentiation marker *KRT6A* and proliferating BC (BC2) in smoke-exposed ALI cultures, whereas the conventional BC1 subset was decreased, supporting basal cell activation by acute smoke exposure (Figure 4i).

In summary, high-resolution transcriptomic profiling revealed cell-type specific alterations of gene expression in response to acute smoke exposure that is dominated by cellular detoxification and inflammatory response. Additionally, acute smoke exposure triggered basal cell activation, proliferation and aberrant differentiation that is associated with an increase of *KRT6A*^+^ basal cells and of pro-inflammatory intermediate cells.

### 2.4. Chronic Smoke Exposure during Cell Differentiation Shifts Basal Cells towards the Squamous Differentiation Associated Phenotype

**Figure 5:**
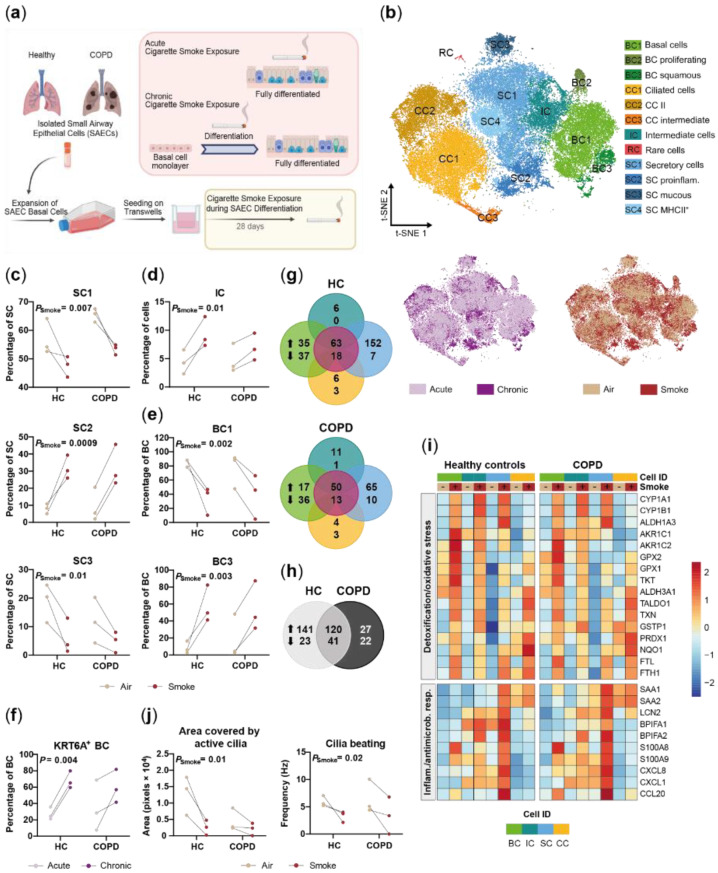
Impact of smoke exposure on differentiating small airway epithelial cells. (**a**) Experimental design to investigate the impact of chronic cigarette smoke exposure throughout differentiation contrasted with the design applied to examine the effect of acute smoke exposure. (**b**) T-SNE plots represent the integrated scRNA-seq data sets of both experiments and depict annotated cell (sub)populations (upper panel) as well as the experimental origin (lower panel, left) and smoking status (lower panel, right) of the cells. (**c**–**e**) Smoke-induced shifts of selected SC and BC subpopulation frequencies and the total IC frequency after chronic smoke exposure throughout differentiation. Data of the respective HC and COPD donors are connected. (**f**) *KRT6A*^+^ BC frequency in SAEC ALI cultures chronically exposed to smoke during differentiation compared to acute smoke exposure. (**g**) Summary of the significantly up- and down-regulated genes (adj. *p* < 0.05, abs. fold change ≥1.5) after chronic smoke exposure across the main SAEC populations (BC, IC, SC and CC according to the color code in (**i**)) of HC and COPD donors (*n* = 3, each). Core genes de-regulated in at least two cell types are summarized in the center (violet circle). (**h**) Comparison of the total gene sets significantly de-regulated by chronic smoke exposure in at least one of the four main cell populations of HC and COPD donors. (**i**) Relative expression of selected genes from the core gene set that are associated with cellular detoxification/oxidative stress and inflammatory/antimicrobial response across the four main SAEC populations. Average gene expression is shown for air control and smoke-exposed (chronic) cells from both, HC and COPD donors. (**j**) Impact of chronic smoke exposure on cilia function as determined by the area covered by active cilia and frequency of cilia beating.

The data suggest that acute CS exposure induces an altered basal cell phenotype and affects basal cell differentiation. To further investigate this hypothesis, SAEC ALI cultures from the same donors were exposed to whole CS throughout the differentiation process (Figure 5a).

ScRNA-seq was performed for air control and smoke-exposed ALI cultures on a total of 26,705 cells (Figure 5b). Integrated analysis on both data sets (acute and chronic) confirmed the previously identified SAEC subpopulations also in the chronic model and therefore an overall conserved SAEC subpopulation architecture (Figure 5b and Figure S2a–c). As observed for the acute setting, CS exposure throughout differentiation significantly altered the frequency of the different SAEC subpopulations with the basal and secretory cell compartments being most strongly affected. A decreased frequency of secretory cell clusters SC1 and SC3 and an increase of pro-inflammatory secretory cells (SC2) and intermediate cells further substantiates an altered basal-to-secretory cell differentiation and increased inflammatory profile upon smoke exposure during differentiation (Figure 5c,d). Additionally, we observed a strong enrichment of basal cells from ALI cultures chronically exposed to smoke during differentiation within the inflammatory and differentiation associated BC3 cluster that was on average 7.4-fold increased across all donors, whereas conventional basal cells (BC1) decreased compared to air controls (Figure 5b,e). Moreover, *KRT6A*^+^ cells were significantly increased within the basal cell population after chronic smoke exposure compared to acute smoking but not the respective air controls, which finally substantiates that basal cells are fundamentally affected by smoke exposure throughout differentiation (Figure 5f and Figure S2d). As observed for acute smoke exposure, shifts of cell subpopulation frequencies were overall similar across HC and COPD donors, suggesting a similar response across both groups (Figure 5c–f).

To further elucidate the impact of smoke exposure during differentiation, also compared to acute smoking, the gene expression profiles of the four main SAEC populations were investigated. Again, the response of COPD- and HC-derived ALI cultures was similar, although more genes were significantly de-regulated in secretory cells from HC than COPD donors (Figure 5g). Gene expression results from secretory cells of COPD and HC showed a significant correlation (R^2^ = 0.84, *p* < 0.0001), indicating an overall similar response which is further substantiated by a strong overlap of smoke-affected gene sets in COPD donors and HC (Figure 5h and Figure S2e). Nevertheless, COPD donors were characterized by an increased expression of the pro-inflammatory gene set that was already observed in the acute model, which further substantiates an inherently increased inflammatory profile of the ALI cultures derived from COPD patients (Figure 3b,c and Table S1).

**Figure 6:**
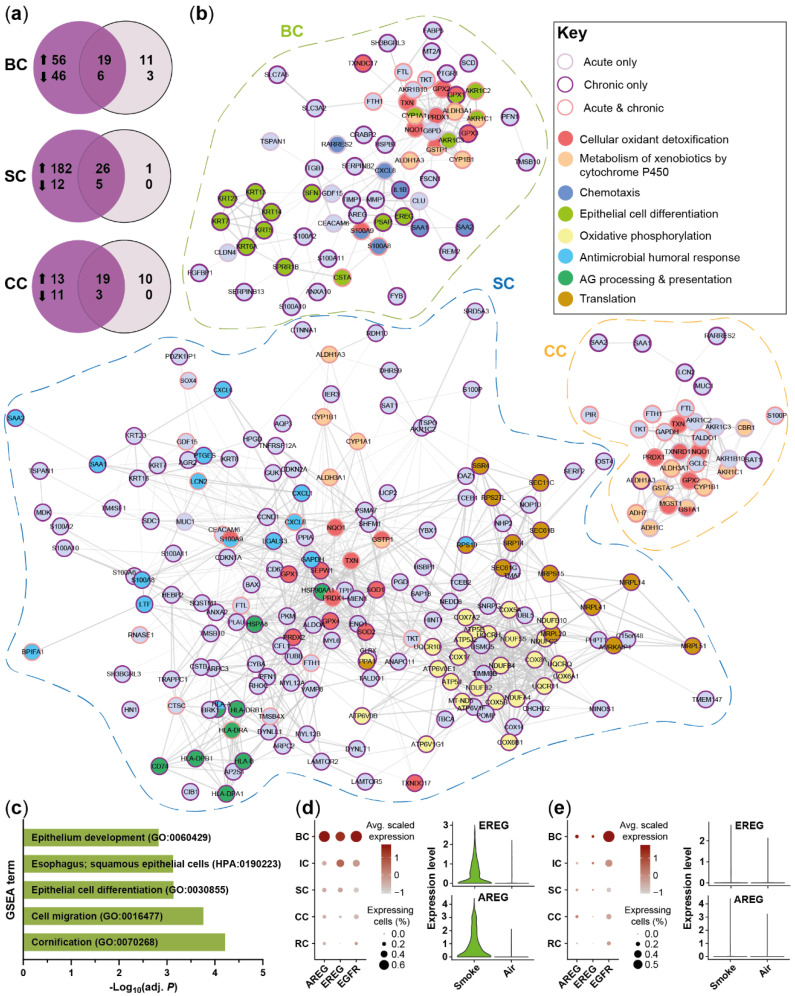
Differentiation of acute and chronic smoke exposure-induced molecular responses. (**a**) Comparison of significantly up- and down-regulated (adj. *p* < 0.05, abs. fold change ≥1.5) genes in BC, SC and CC after acute smoke exposure and smoke exposure throughout differentiation compared to air controls. (**b**) Functional network analysis based on the genes up-regulated by acute smoke exposure and/or smoke exposure throughout differentiation in BC, SC and CC. Node (gene) borders indicate whether the respective gene is up-regulated by acute or chronic smoke exposure or both. Node colors refer to selected molecular mechanisms that are significantly associated (adj. *p* < 0.05) with the gene set of the respective cell types. (**c**) Selected molecular processes identified by GSEA that are significantly (adj. *p* < 0.05) associated with the gene set up-regulated in BC only after chronic smoke exposure compared to air controls. (**d**,**e**) Relative expression of the EGFR-signaling associated genes *AREG*, *EREG* and *EGFR* across SAEC populations after chronic (**d**) and acute (**e**) smoke exposure is illustrated by dot plots. Comparison of normalized *EREG* and *AREG* expression in basal cells from smoke-exposed and air control ALI cultures is represented by violin plots.

The core gene set induced by smoke exposure in at least two of the four main cell types was associated with an increased cellular detoxification, oxidative stress (*CYP1A1, CYP1B1, TXN*, *PRDX1*, *GPX1, GPX2*, *GSTP1*, *GCLC*, *TKT*, *NQO1*, *MGST1, FTL, FTH*, *AKR1C1, AKR1C2, ALDH1A3, ALDH3A1*) and inflammatory response (*SAA1, SAA2, S100A8/9, CXCL8, CXCL1, CCL20*) (Figure 5g,i). Intermediate and ciliated cells were mainly characterized by a de-regulated expression of genes included in the core gene set whereof the vast majority was also induced upon acute smoke exposure (Figure 4d). Both, acute and chronic smoke exposure induced a similar transcriptional response in ciliated cells that is dominated by a cellular detoxification and oxidative stress response and few inflammatory/antimicrobial markers after chronic smoke exposure (*SAA1, SAA2, LCN2, RARRES2, MUC1*) (Figure 4d, Figure 5i and Figure 6b). However, impaired ciliary function characterized by a reduced area covered by actively beating cilia and a reduced cilia beat frequency was only observed after chronic but not acute smoke exposure (Figure 5j and Figure S1e).

In contrast to intermediate and ciliated cells, secretory and basal cells were characterized by cell-type specific de-regulation of several genes (Figure 5g). To better understand these cell type-specific effects, also in comparison to acute smoke exposure, a network analysis on the de-regulated gene sets of these cell types from HC was performed (Figure 6). Secretory cells were more strongly affected by chronic than acute smoke exposure when compared to air controls (Figure 6a). Chronic smoke exposure induced the expression of oxidative phosphorylation (e.g., *COX5A, NDUFB2, ATP5J, UQCRQ*) and translation associated gene modules, including mitochondrial ribosome (*MRPS15, MRPL20, MRPL51*) and endoplasmic reticulum processing and transport (*SEC61B, SEC11C, SRP14, SSR4*) associated genes (Figure 6b). Additionally, we observed a specific or increased induction of immunity related molecular processes, including antigen processing and presentation (*HLA-DRA, HLA, DRB1, HLA, HLA-B*) as well as antimicrobial and immune cell recruitment associated genes (*LTF, S100A8, CXCL6, CXCL1, SAA1, SAA2*) (Figure 6b). Collectively, these data suggest an increased cellular activity, energy demand and inflammatory response in secretory cells after chronic than acute smoke exposure. As observed for secretory cells, more genes were found to be differentially expressed in basal cells after chronic compared to acute smoke exposure, which is consistent with the increase of *KRT6A*^+^ basal cells (Figure 5f and Figure 6a). Acute and chronic smoke exposure induced the expression of pro-inflammatory genes and genes that are implicated in immune cell recruitment (*CXCL8, RARRES2, IL1B, SAA1, SAA2, S100A8/9*) although the de-regulated gene sets differed from each other (Figure 6b). In contrast, several keratins associated with epithelial cell and squamous differentiation (*KRT6A, KRT13*, *KRT14)* and *SPRR1B* [3,35,36,52] were selectively induced after chronic smoke exposure, indicating the onset of basal cell hyperplasia and squamous metaplasia (Figure 6b). GSEA on the gene set that was induced after chronic but not after acute smoke exposure corroborated the transformation into metaplasia associated basal cells by significantly enriched molecular processes that are linked to epithelial cell differentiation and squamous epithelial cells (Figure 6c). Concomitantly, basal cells showed a selectively increased expression of the EGFR ligands *AREG* and *EREG* after chronic smoke exposure, indicating autocrine EGFR signaling in basal cells (Figure 6d,e). Beyond basal cells, *EGFR* expression was also detected in the remaining SAEC populations, suggesting that basal cell-derived EGFR ligands also affect the entire SAEC population (Figure 6d,e).

Taken together, these analyses confirmed the previously identified SAEC subpopulations in both smoke exposure models and further substantiated an altered basal cell differentiation upon smoke exposure. Smoke exposure throughout differentiation provoked a fundamental transformation of basal cells towards a squamous metaplasia associated phenotype that was identified as important regulator of smoke-induced inflammatory and remodeling associated signaling.

### 2.5. Smoking Affects the Expression of SARS-CoV-2 Entry Factors

Recent studies suggest that smokers are more susceptible to severe SARS-CoV-2 infections compared to non-smokers [53,54]. Considering the continuing pandemic and need for relevant model systems to examine the impact of smoke exposure on viral infection the expression of SARS-CoV-2 entry factors was investigated in smoke-exposed and air control ALI cultures.

**Figure 7:**
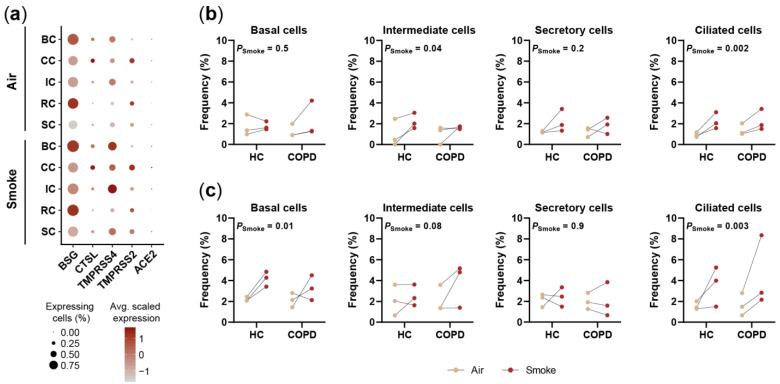
Smoke exposure affects the expression of SARS-CoV-2 entry factors in SAEC populations. (**a**) Dot plot illustrating the average relative expression and fraction of cells that express genes encoding for SARS-CoV-2 entry factors across the main SAEC populations. (**b**,**c**) Smoke induced shifts of *ACE2*^+^ cell frequency in SAEC ALI cultures from HC and COPD donors (*n* = 3, each). Effects are shown for acute (**b**) and chronic (**c**) smoke exposure.

Several genes that have been linked with SARS-CoV-2 infection were found to be expressed in the SAEC ALI cultures including *BSG* (CD147), *CTSL*, *TMPRSS4*, *TMPRSS2* and *ACE2* (Figure 7a) [55,56,57]. Among those genes *BSG* showed a strong expression across all cell types of air control and smoke-exposed ALI cultures. In contrast, *CTSL, TMPRSS4* and *TMPRSS2* were characterized by overall lower and cell type specific expression patterns but all of them were found in ciliated cells (Figure 7a). *ACE2* was detected in only a minor fraction (<10%) of the cells across all cell types (Figure 7a). These characteristic expression profiles across cell types were similar for both model systems (Figure S3).

Smoke variably increased the fraction of cells expressing the SARS-CoV-2 infection associated genes (Table S2). Among the investigated genes, the strongest effects were observed for *TMPRSS4,* which is reflected by a significant increase (*p* < 0.05) of basal, intermediate, secretory and ciliated cells expressing the gene after acute and chronic smoke exposure (Figure 7a, Figure S3 and Table S2). Beyond *TMPRSS4*, angiotensin converting enzyme 2 (ACE2) expression was investigated in closer detail since ACE2 was repeatedly shown to serve as SARS-CoV-2 entry receptor [58,59]. *ACE2* was detected in <4% of the cells per SAEC population in air controls and showed cell type-specific induction after acute and chronic smoke exposure (Figure 7a–c). Importantly, ciliated cells showed a consistent, on average 1.9-fold and 2.5-fold increase of *ACE2*^+^ cells across all donors upon acute and chronic smoke-exposure, respectively (Figure 7b,c).

In summary, we observed variable induction of SARS-CoV-2 entry factors and a consistent increase of *ACE2*^+^ ciliated cells in two independent experiments after acute and chronic smoke exposure, suggesting that CS exposure might lead to an increased susceptibility for SARS-CoV-2 infections. These observations further illustrate the power of this study, which allows unbiased investigation of CS-induced molecular responses across the different SAEC populations at single-cell resolution, making these data a valuable resource for various areas in respiratory research.

## 3. Discussion

This study was performed to establish a comprehensive cell atlas of the in vitro differentiated small airway epithelium and allows novel insights into the normal and smoke-modulated SAEC landscape including cell type-specific responses to CS exposure. ScRNA-seq identified 12 cell clusters spanning all known airway epithelial cell populations (basal, intermediate, secretory, ciliated and tuft-like/brush cells as well as pulmonary ionocytes), demonstrating that the main characteristics of human SAEC communities were reflected in the SAEC ALI cultures [3,33,60]. Our data provide novel information on specialized SAEC subpopulations and states. Beyond the known mucous secretory cell (SC3) subset [33], this study resolved a *SCGB3A2*^+^ secretory cell cluster (SC4) with low mucin-related gene expression that has recently been observed in human lung specimens. However, these studies were not able to explicitly demonstrate its presence in the small airways [61,62]. Diversity in the secretory cell fraction was further demonstrated by variable expression of MHC-II components. Although MHC-II expression by airway epithelial cells and their ability to stimulate T-cell proliferation was reported decades ago [63,64], this study suggests functional heterogeneity within the MHC-II^Hi^ secretory cell fraction including subsets with anti-inflammatory (SC4, *SCGB3A2*^+^) and pro-inflammatory properties (SC2). These observations indicate that the small airway secretory cell population is composed of different specialized subpopulations including two subsets that may contrarily modulate the immune response in the small airways.

Smoking is a risk factor of many lung diseases, including COPD [9,10], and known to affect gene expression in the small airway epithelium [30,31]. However, the impact of CS on SAEC populations is not yet fully understood. Here, scRNA-seq allowed the differentiation of acutely and chronically smoke-induced molecular responses on cell type resolution. Cell-type specific induction of oxidative stress and xenobiotic metabolism responses was previously reported in airway epithelial cells from smokers, indicating that these processes are reflected in our model systems [3,32,33]. This study identified these molecular responses as fast and stable protective measures to counteract smoke-induced damage of the small airway epithelium, whereas the induction of inflammatory chemokine signaling is increased in response to chronic smoke, particularly in basal cells.

The data also provide insights into smoke-induced cellular dynamics that reveal new perspectives on pathological processes seen in smokers and COPD patients. Smoke-induced impaired basal cell differentiation as observed in this study and previously in epithelial cells [65] may contribute to an inflammatory environment and immune cell recruitment through the accumulation of pro-inflammatory basal-to-secretory intermediates. Moreover, the associated decline of the MUC5B/PIGR^Hi^ secretory cell subset may directly contribute to the development of COPD. This is corroborated by previous studies that showed decreased *PIGR* levels and impaired host defense upon chronic smoke exposure [65], development of COPD-like pathology in polymeric immunoglobulin receptor (pIgR) deficient mice [66] and decreased pIgR levels in COPD patients, importantly in incompletely differentiated and squamous metaplasia dominated airway epithelial regions [24,67].

Recently, Rao et al. [68] reported a massive increase of a metaplasia associated KRT5/p63^+^ airway progenitor cell subset in end-stage and moderate COPD. This subset was characterized by the expression of pro-inflammatory chemokines including *CXCL8* and *CCL20* and was shown to give rise to squamous cell metaplasia and neutrophil recruitment [68]. However, mechanisms underlying the expansion of the pro-inflammatory and metaplastic progenitor subset in COPD patients could not be elucidated by Rao and colleagues [68]. The squamous basal cell subset (BC3) identified in this study was characterized by a similar profile including the expression of the same neutrophil chemoattractants (*CXCL8, CCL20*) and represented the major source of the EGFR ligands amphiregulin (*AREG*) and epiregulin (*EREG*). *AREG* was shown to be induced by CS in consequence of squamous basal cell differentiation and to promote basal cell and mucous hyperplasia as well as cilia shortening in ciliated cells [35]. These observations suggest that the BC3 subset is a main regulator of those smoke-induced alterations in the ALI model systems, thereby contributing to initial stages of basal and goblet cell hyperplasia which are critical hallmarks of COPD [13,14,22,23,24,25]. As observed for the metaplastic subset in vivo [68], the squamous phenotype was rarely detected in most air control and acute smoke-exposed ALI cultures but massively increased after chronic smoke exposure throughout differentiation. This study provides evidence that chronic CS stimulates the accumulation of a COPD-relevant squamous and pro-inflammatory basal cell phenotype, most likely in response to smoke-induced injury which is supported by an impaired epithelial barrier integrity and increased expression of wound repair associated markers (*KRT6A*, *FN1*, *VIM*) including *EREG* by this basal cell subset [69,70,71].

In previous studies we demonstrated that some COPD-relevant morphological, functional and transcriptional properties are conserved in COPD-derived SAEC ALI cultures [34]. This study confirms an increased expression of some COPD-associated inflammatory markers (*CCL20*, *LCN2*, *SAA1*, *SAA2*) [72,73,74], while providing cellular resolution for their expression. The cellular resolution of scRNA-seq also revealed increased baseline levels of the BC3 subset in ALI cultures of a COPD patient, which reinforces the link between this smoke-induced basal cell subset in COPD pathology and demonstrates that it can be maintained in ALI culture; an assumption that is also shared by others [68].

The current study is based on data from three healthy donors and three COPD donors. A higher donor number might increase the statistical power for the comparison between HC- and COPD-derived cultures and between smoke and air-treated cultures. Nevertheless, our model systems consistently recapitulate smoke-induced processes and cell subpopulations seen in vivo in two independent experiments. Our data also show that CS exposure drives pathological remodeling in the small airway epithelium seen in COPD, identifies a squamous basal cell population that appears to be an important regulator of the remodeling and inflammatory processes and reveals a previously unrecognized heterogeneity within the small airway secretory cell landscape that is also affected by smoke. Finally, broader relevance of the SAEC ALI smoke models was demonstrated by revealing smoke-dependent variable induction of various SARS-CoV-2 entry factors (including *ACE2*) across cell types. Increased *ACE2* expression levels have been previously reported for our SAEC ALI model system following chronic CS exposure throughout differentiation [53], which further substantiates the findings of the current study. This study now provides the cellular resolution to evaluate the contribution of the different cell types to the increased *ACE2* expression levels seen before in bulk [53]. Data from the smoked ALI model are also consistent with previous findings showing increased expression levels in human smokers [53].

Being aware of the SAEC landscape and the cell (sub)type-specific roles in response to smoke exposure may contribute to the development of cell-specific therapeutic approaches for example in the context of COPD.

## 4. Materials and Methods

### 4.1. Small Airway Epithelial Cell Air–Liquid Interface Culture

Human small airway epithelial cells (#CC-2547 and #CC-2934, Lonza, Basel, Switzerland) of three healthy donors and three COPD patients (Table S3) were cultured as described previously [34]. As stated by Lonza, the cells were isolated from donated human tissue after obtaining permission for their use in research applications by informed consent or legal authorization. SAECs were grown in PneumaCult™-Ex Plus Medium (Stemcell Technologies, Vancouver, Canada) on rat tail collagen type 1 (Corning Life Sciences B.V., Amsterdam, the Netherlands) coated transwells (#3460, Corning Life Sciences B.V., Amsterdam, the Netherlands) until confluent. Cells were further cultivated in air–liquid interface using Pneumacult-ALI-S Medium (Stemcell Technologies, Vancouver, Canada) for four weeks until fully differentiated.

### 4.2. Cigarette Smoke Exposure

SAECs were exposed to whole CS of 3R4F reference cigarettes (University of Kentucky, Lexington, KY, USA) using an automated cigarette smoking machine (In-Expose smoking robot, Scireq Montreal, QC, Canada) and the P.R.I.T.^®^ (Professional In-Vitro Technologies) ExpoCube ^®^ in the experimental setup developed by Fraunhofer Institute for Toxicology and Experimental Medicine as described previously [34]. For the acute smoke exposure model, fully differentiated SAECs were exposed to CS on four consecutive days. For the smoke exposure upon differentiation, SAECs were exposed to CS three times a week during the differentiation phase (28 days) beginning on day 0 (= day of air-lift). Four cigarettes were smoked in parallel in compliance to ISO 3308, drawing every 15 s a puff from the sequentially smoked cigarettes (9 puffs per cigarette). The smoke was diluted with ambient air (dilution rate: 0.5 l/min). Readouts were performed 24 h post last exposure.

### 4.3. Preparation of Single-Cell Suspensions

Small airway epithelial cell ALI cultures grown on transwell inserts were enzymatically and mechanically dissociated. The SAEC ALI transwell inserts were first incubated in 1.5 mL of Accumax solution (Sigma-Aldrich, St. Louis, MO, USA) for 15 min at room temperature. 300 µL of Accumax solution were removed from the apical side of the ALI culture and stored in a gentleMACS C Tube on ice until further processing. Another 300 µL of fresh Accumax solution were added to the apical side of the transwell insert and incubated for an additional 15 min. Cells were detached from the transwell membrane by gentle tapping of the plate and the membrane was rinsed with Accumax solution to wash off any remaining cells. Washed-off cells were transferred to the gentleMACS C Tube in order to dissociate the cells by the gentleMACS Dissociator (Miltenyi Biotec, Bergisch Gladbach, Germany) using the default m_lung_01 program. Following dissociation, single-cell suspensions were kept on ice throughout the procedure. Cells were centrifuged (4 °C) and gently resuspended in HBSS/0.04% bovine serum albumin buffer. Finally, resuspended cells were filtered through a 30 µm cell strainer. Cell concentration and viability were determined using a NucleoCounter NC-200^TM^ (ChemoMetec, Allerod, Denmark). Residual dead cells were removed using a Dead Cell Removal Kit (Miltenyi Biotec, Bergisch Gladbach, Germany).

### 4.4. Single-Cell RNA-Sequencing

#### 4.4.1. Library Preparation and Sequencing

ScRNA-seq libraries were prepared using the Chromium Controller and Single Cell 3′ Reagent Kits v2 (10x Genomics, Pleasanton, CA, USA) according to the manufacturer’s instructions. Briefly, 8700 cells were loaded onto the Single Cell Chip A for single-cell encapsulation into nanodroplets and the cDNA was amplified by 13 PCR cycles. Sequencing libraries were prepared from 150 ng of cDNA and amplified by 13 PCR cycles during the final index PCR reaction. Libraries were purified with a double-sided size selection as described by the manufacturer. An additional clean-up was performed with one volume of SPRISelect Beads (Beckman Coulter, Brea, CA, USA) to ensure complete removal of adapter and primer dimers. Purified libraries were on average 437 bp and sequenced on a HiSeq 4000 (Illumina, San Diego, CA, USA) to an average read depth per cell of approximately 50,000 reads and 80,000 reads for the acute and chronic model, respectively. Read 1 of the paired-end sequencing comprised 26 bp (16 bp cell barcode and 10 bp UMI), whereas 8 bp and 98 bp were sequenced for the index read and read 2 (transcript sequence read), respectively. Cluster generation was performed by the cBot system (Illumina, San Diego, CA, USA) using a HiSeq3000/4000 PE Cluster Kit (Illumina, San Diego, CA, USA).

#### 4.4.2. Data Processing

Sequencing data were processed by Cell Ranger v2.1.1 (10x Genomics, Pleasanton, CA, USA) to generate UMI-based gene count matrices per sample. The pipeline made use of bcl2fastq v2.17.1.14 (Illumina, San Diego, CA, USA) for demultiplexing and STAR v2.5.1b [75] for read alignment. Sequencing reads were aligned to the GRCh38 reference genome and annotated according to Ensembl release 86.

#### 4.4.3. Quality Control, Cluster Analysis and Visualization of scRNA-seq Data

Filtering for high quality cells as well as data normalization and scaling were performed by the Seurat R package v2.2.1 [76]. Cells expressing 500 to 6000 genes were kept for downstream analysis. Additionally, cells with >20% of mitochondrial transcripts as well as genes that were expressed in less than three cells were removed from subsequent analysis. UMI counts of the filtered cells were normalized per sample by dividing the gene counts per cell by its total count, then multiplied by 10,000 and log transformed. Subsequently, data were scaled and centered according to Seurat’s defaults using the ScaleData function. Additionally, linear regression was performed to account for differences in the UMI count per cell.

For downstream analysis of the acute smoke model data, canonical correlation analysis was performed on a gene set of highly variable genes using the RunMultiCCA function. This gene set comprised genes that were observed in at least two data sets within the 800 most variable genes. The Seurat AlignSubspace function was applied to project data sets of the different samples onto a common subspace while aligning them by donor. The 15 canonical vectors with the most variance were used for alignment and data sets were integrated into one R Data object. Subsequent cluster analysis to identify cell populations in the SAEC samples was performed using the shared nearest neighbor modularity optimization-based algorithm as implemented in the FindClusters function of the Seurat package. Single-cell clustering was performed on the top 15 canonical vectors and 0.5 resolution. Cell clusters were visualized by t-distributed stochastic neighbor embedding (t-SNE) plots as implemented in the Seurat package. Quality control of the cell clusters identified a cell cluster with a low median UMI count of 1748, which is 6.3-fold lower than the median UMI count of all remaining cells (11,085). This low-quality cell cluster accounted for 0.7% of all cells and was removed from subsequent analyses.

Integrated analysis of the acute and chronic smoke model data was performed as described above using the first 28 canonical vectors for alignment and cluster analysis at 0.5 resolution. Two clusters representing 0.9% and 0.2% of all cells were identified as low-quality cells that were characterized by a low median UMI count of 1735 and 895, which were 6.6- and 12.8-fold lower than the median UMI count of the remaining cells (11,461). Both low-quality clusters were removed for further analysis.

#### 4.4.4. Differential Expression Analysis

Differentially expressed genes were identified using the MAST linear model approach v1.4 [77]. Genes expressed in at least 10% of the cells in one of the compared clusters or groups were included in differential gene expression analysis. Genes at least 1.5-fold de-regulated were considered to be significantly differentially expressed if *p*-values adjusted for multiple testing were <0.05 (Bonferroni correction).

#### 4.4.5. Gene Set Enrichment and Protein–Protein Interaction Network Analysis

GSEA was performed via the g:Profiler web server [78] using default settings. Protein–protein interaction networks were generated by the STRING database v11.0 [79] using the default settings and visualized using Cytoscape v3.8.2 [80].

#### 4.4.6. Pseudotime Analysis

Cells from healthy donors were extracted from the Seurat object and converted into a CellDataSet object for pseudotime analysis using Monocle v2.6.3 [81]. Firstly, genes with a high dispersion (median expression ≥0.1 and empirical dispersion ≥ estimated value as determined by the mean-variance model) were identified via the dispersionTable function. Next, dimensionality reduction was performed by means of the reduceDimension function and the DDRTree method to reduce the space to two dimensions. Cells were then ordered along the trajectory using the orderCells function and specifying the segment containing most BC1 cells as the root of the trajectory. Genes that were differentially expressed along pseudotime were identified by the differentialGeneTest function and their expression along pseudotime was visualized by the plot_genes_in_pseudotime function.

### 4.5. Transepithelial Electrical Resistance

To assess epithelial barrier integrity TEER was monitored using an EVOM2 epithelial volt-ohmmeter (World precision Instruments, Sarasota, FL, USA) according to the manufacturer’s protocol as described previously [34]. Phosphate buffered saline (300 µL) was added to the apical compartment of the insert to enable resistance measurement. The measured resistance was multiplied by the surface area of the epithelium (1.12 cm^2^) to obtain TEER (Ω*cm^2^).

### 4.6. Flow Cytometry

Flow cytometry analysis was performed as described previously [34]. Shortly, single cell suspensions of SAEC ALI cultures were fixed in 4% paraformaldehyde for 20 min at room temperature. Cells were washed twice using Perm/Wash Buffer (BD Biosciences, San Jose, CA, USA) and blocked to prevent non-specific binding using Human BD Fc Block (BD Biosciences, San Jose, CA, USA). Cells were stained in Perm/Wash Buffer for 1 h at room temperature and analyzed using a BD LSRFortessa X-20 cytometer equipped with DIVA-software (BD Biosciences, San Jose, CA, USA).

Following antibodies were used for staining; Rabbit monoclonal Anti-Cytokeratin 5 antibody, clone EP1601Y, ab193895 (Abcam, Cambridge, UK), mouse monoclonal Anti-MUC5AC antibody, clone 45M1, NBP2-32732AF405 (Novus Biologicals, Centennial, CO, USA), rat monoclonal Anti-SCGB1A1 antibody, Clone #394324, MAB4218 (R&D; Minneapolis, MN, USA), rat monoclonal Anti-SCGB1A1 antibody, Clone #394324, MAB4218 (R&D; Minneapolis, MN, USA).

### 4.7. Histology

SAEC ALI cultures growing on transwell membranes were fixed in 4% paraformaldehyde overnight and embedded in paraffin. Blocks were sectioned in 3–4 µm thick slices. Paraffin-embedded tissue slides were subjected to heat-induced antigen retrieval in citrate buffer (pH 6.0, H.I.E.R, BioLegend San Diego, CA, USA) for 30 min. Mouse monoclonal Anti-MUC5AC antibody, clone 45M1, NBP2-32732AF488 (Novus Biologicals, Centennial, CO, USA) was used for immunofluorescence antigen detection. Slides were mounted in ProLong™ Diamond Antifade Mountant (Thermo Fisher Scientific, Waltham, MA, USA) and stored in the dark. Images were taken using the Zeiss Laser Scanning Microscope 710 (Carl Zeiss, Oberkochen, Germany).

### 4.8. Statistical Analysis

Cilia beating data were analyzed by repeated measures Two-Way ANOVA. Non-parametric analysis of variance from cell (sub)population frequencies and TEER data was performed by the Aligned Rank Transform procedure using the ARTool R package v0.10.8, which allows factorial non-parametric analysis and handles repeated measure data [82].

### 4.9. Data Visualization

Violin plots, dot plots and t-SNE plots were generated by the respective Seurat functions and heatmaps via the pheatmap R package v1.0.8. Protein–protein interaction networks were generated by the STRING database webpage and the layout was manually adapted for publication. Bar plots and before and after graphs were generated using GraphPad Prism v8.3.0 (GraphPad Software, San Diego, CA, USA). Experimental design overviews were created with Biorender.com.

## Data Availability

ScRNA-seq data are available in the European Nucleotide Archive database under accession code PRJEB44878.

## Supplementary Materials

The following are available online at https://www.mdpi.com/article/10.3390/ijms22147646/s1.
