# Supplementary material for: Cigarette Smoke Specifically Affects Small Airway Epithelial Cell Populations and Triggers the Expansion of Inflammatory and Squamous Differentiation Associated Basal Cells
Source: Int J Mol Sci. 2021 Jul 16;22(14):7646. doi: 10.3390/ijms22147646 (PMC8305830; doi:10.3390/ijms22147646)
Supplement: Supplementary file 1 [file ijms-22-07646-s001.zip › ijms-1286159-supplementary.pdf]

Supplementary Information

Cigarette smoke specifically affects small airway epithelial cell populations and triggers the expansion of inflammatory and squamous differentiation associated basal cells

Christian T. Wohnhaas, Julia A. Gindele, Tobias Kiechle, Yang Shen, Germán G. Leparc, Birgit Stierstorfer, Heiko Stahl, Florian Gantner, Coralie Viollet, Jürgen Schymeinsky, Patrick Baum

Supplementary Figure Legends

Figure S1. Acute smoke model.

(**a**) T-SNE plot illustrating the SAEC (sub)populations identified in the integrated scRNA-seq data set of the smoke model and the same t-SNE plot coloured by donor (HC: donors A-C, COPD: donors D-E; bottom panel). (**b**) Normalized *MUC5AC* expression level visualized per individual cell. (**c**) Relative expression and fraction of cells that express pulmonary ionocyte (*ASCL3*, *CFTR*, *TFCP2L1*, *CEL*, *CLCNKB*), tuft-like cell (*POU2F3*, *HCK*, *LRMP*, *NREP*) and pulmonary neuroendocrine cell (*CHGA*, *ASCL1*, *GRP*, *CALCA*) marker genes across the cell (sub)populations. (**d**) Frequency of the identified SAEC (sub)populations in smoke-exposed and air control SAEC ALI cultures per donor. (**e**) Impact of acute smoke exposure on cilia function as determined by the area covered by active cilia and frequency of cilia beating.

Figure S2. Chronic smoke model.

(**a**) T-SNE plot illustrating the SAEC (sub)populations identified in the integrated scRNA-seq data set of the chronic and acute smoke models (upper panel) and the same plot coloured by smoke model, smoking status, disease status and donor (lower panel). (**b**) Relative expression and fraction of cells that express BC (*KRT5*, *KRT15*), CC (*FOXJ1*, *DNAI1*), SC (*CYP2F1*, *SCGB1A1*), RC (*FOXI1*, *ASCL3*) marker genes and genes that are strongly expressed by IC as well as BC or SC (*AQP3*, *MIR205HG*, *TSPAN8*, *C3*) across the cell clusters of the chronic smoke model. (**c**) Heatmaps depict relative expression levels of genes across CC, BC and SC subpopulations of the chronic smoke model that were significantly differentially expressed (adj. $P < 0.05$, abs. fold change (FC) ≥ 1.5) between the respective subpopulations of the acute smoke model (compare Fig. 2f). (**d**) $KRT6A^{+}$ BC frequency in air control SAEC ALI cultures of the chronic and acute model systems. Data are connected by donor. (**e**) Correlation of genes differentially expressed by chronic smoke exposure in SC of HC and/or COPD donors based on their FC compared to air controls.

Figure S3. Expression of SARS-CoV-2 entry associated genes across SAEC populations.

Dot plots illustrate the average relative expression and fraction of cells that express genes encoding for SARS-CoV-2 entry factors across the main SAEC populations of the acute and chronic model system. Data are shown for smoke exposed and air control cells.

Figure S1

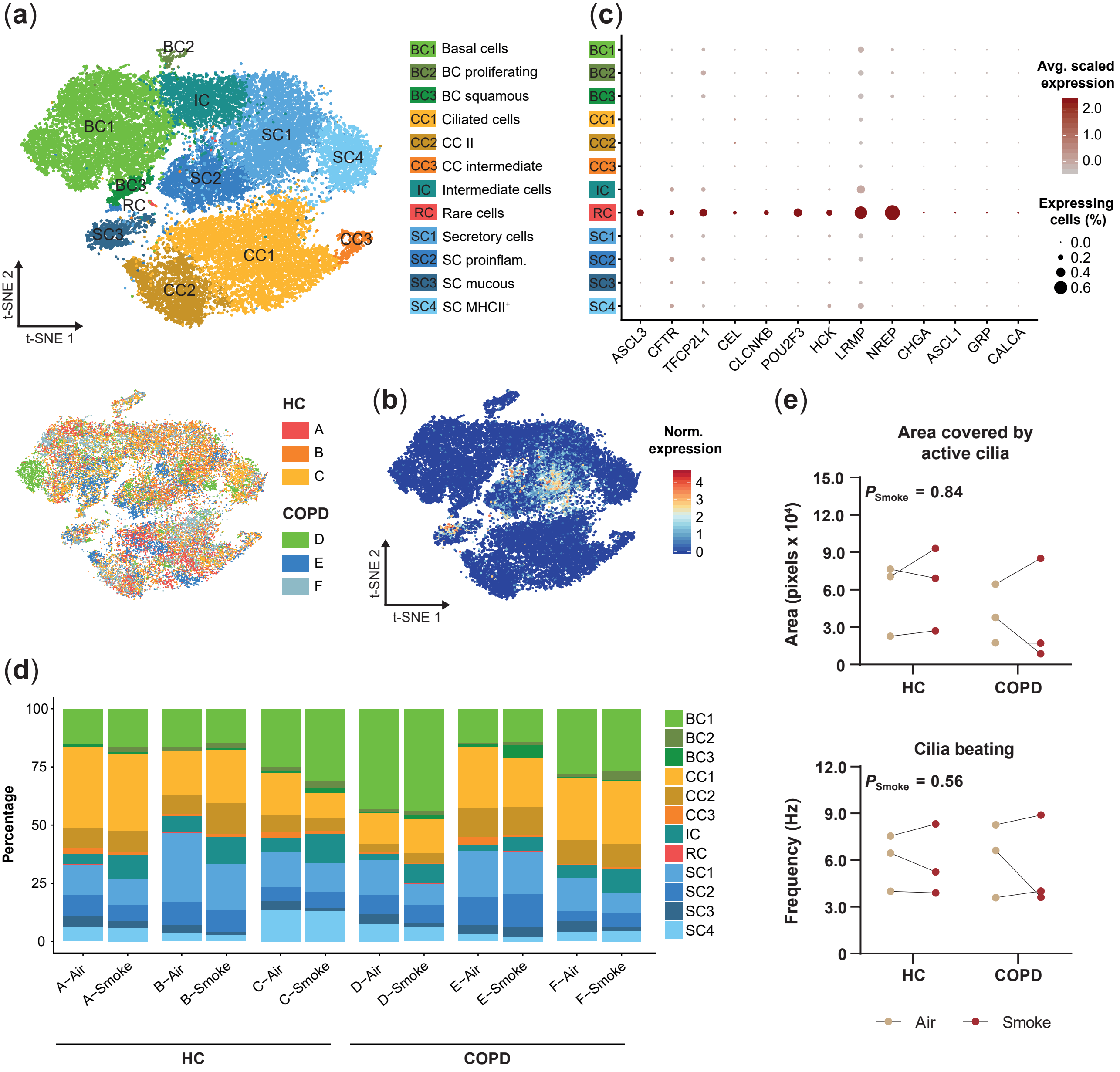

(a)
BC2
IC
BC1
SC1
SC4
BC3
SC2
RC
SC3
CC3
CC1
CC2
t-SNE 2
t-SNE 1
BC1 Basal cells
BC2 BC proliferating
BC3 BC squamous
CC1 Ciliated cells
CC2 CC II
CC3 CC intermediate
IC Intermediate cells
RC Rare cells
SC1 Secretory cells
SC2 SC proinflam.
SC3 SC mucous
SC4 SC MHCII⁺
HC
A
B
C
COPD
D
E
F
(b)
Norm. expression
4
3
2
1
0
t-SNE 2
t-SNE 1
(c)
Avg. scaled expression
2.0
1.0
0.0
Expressing cells (%)
0.0
0.2
0.4
0.6
ASCL3
CFTR
TFCP2L1
CEL
CLCNKB
POU2F3
HCK
LRMP
NREP
CHGA
ASCL1
GRP
CALCA
(d)
Percentage
100
75
50
25
0
A–Air
A–Smoke
B–Air
B–Smoke
C–Air
C–Smoke
D–Air
D–Smoke
E–Air
E–Smoke
F–Air
F–Smoke
HC
COPD
(e)
Area covered by active cilia
P_Smoke = 0.84
Area (pixels x 10⁴)
15.0
12.0
9.0
6.0
3.0
0
HC
COPD
Cilia beating
P_Smoke = 0.56
Frequency (Hz)
12.0
9.0
6.0
3.0
0
HC
COPD
Air
Smoke

Figure S2

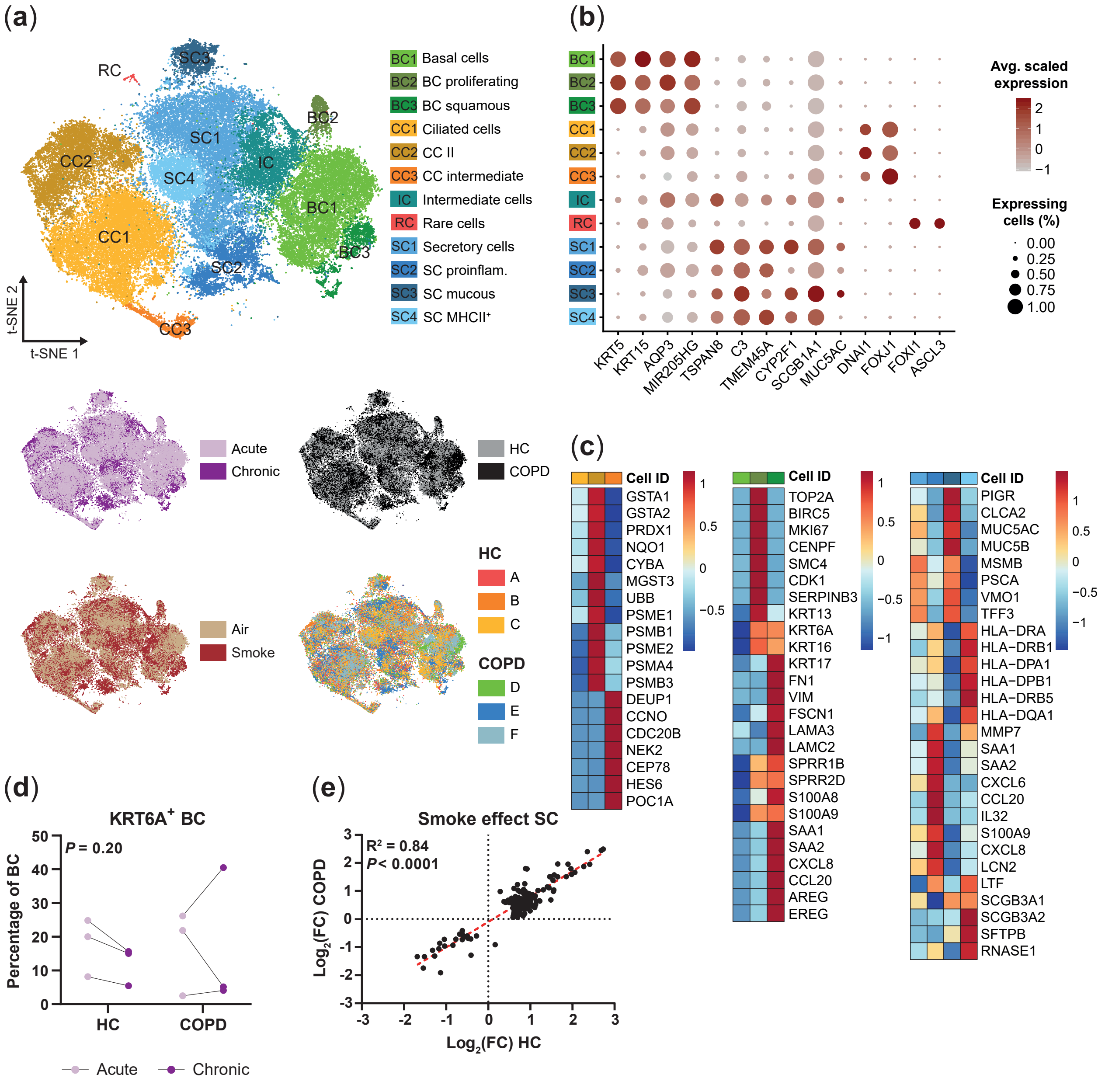
(a)
RC
SC3
BC2
SC1
CC2
IC
SC4
BC1
CC1
BC3
SC2
CC3
t-SNE 2
t-SNE 1
BC1 Basal cells
BC2 BC proliferating
BC3 BC squamous
CC1 Ciliated cells
CC2 CC II
CC3 CC intermediate
IC Intermediate cells
RC Rare cells
SC1 Secretory cells
SC2 SC proinflam.
SC3 SC mucous
SC4 SC MHCII+
(b)
Avg. scaled expression
2
1
0
−1
Expressing cells (%)
0.00
0.25
0.50
0.75
1.00
KRT5
KRT15
AQP3
MIR205HG
TSPAN8
C3
TMEM45A
CYP2F1
SCGB1A1
MUC5AC
DNAI1
FOXJ1
FOXI1
ASCL3
Acute
Chronic
HC
COPD
Air
Smoke
HC
A
B
C
COPD
D
E
F
(c)
Cell ID
GSTA1
GSTA2
PRDX1
NQO1
CYBA
MGST3
UBB
PSME1
PSMB1
PSME2
PSMA4
PSMB3
DEUP1
CCNO
CDC20B
NEK2
CEP78
HES6
POC1A
1
0.5
0
−0.5
Cell ID
TOP2A
BIRC5
MKI67
CENPF
SMC4
CDK1
SERPINB3
KRT13
KRT6A
KRT16
KRT17
FN1
VIM
FSCN1
LAMA3
LAMC2
SPRR1B
SPRR2D
S100A8
S100A9
SAA1
SAA2
CXCL8
CCL20
AREG
EREG
1
0.5
0
−0.5
−1
Cell ID
PIGR
CLCA2
MUC5AC
MUC5B
MSMB
PSCA
VMO1
TFF3
HLA−DRA
HLA−DRB1
HLA−DPA1
HLA−DPB1
HLA−DRB5
HLA−DQA1
MMP7
SAA1
SAA2
CXCL6
CCL20
IL32
S100A9
CXCL8
LCN2
LTF
SCGB3A1
SCGB3A2
SFTPB
RNASE1
1
0.5
0
−0.5
−1
(d)
KRT6A+ BC
P = 0.20
Percentage of BC
50
40
30
20
10
0
HC
COPD
Acute
Chronic
(e)
Smoke effect SC
R² = 0.84
P< 0.0001
Log2(FC) COPD
Log2(FC) HC
3
2
1
0
-1
-2
-3

Figure S3

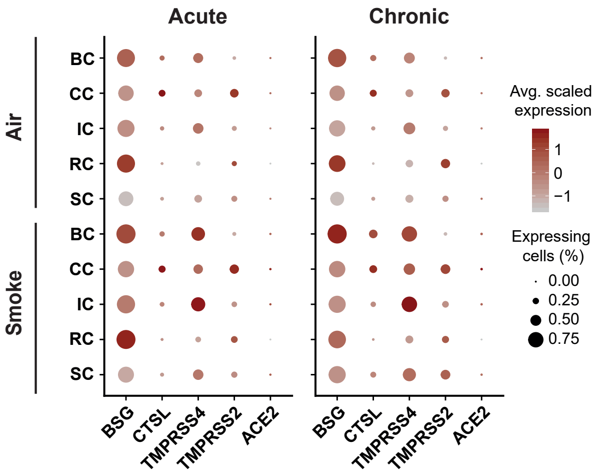

Acute
Chronic
Air
Smoke
BC
CC
IC
RC
SC
BSG
CTSL
TMPRSS4
TMPRSS2
ACE2
Avg. scaled expression
1
0
−1
Expressing cells (%)
0.00
0.25
0.50
0.75

Supplementary Tables

Table S1. Pro-inflammatory genes differentially expressed between COPD and HC donors of the chronic smoke model.

Genes	Basal cells		Ciliated cells		Secretory cells		Intermediate cells	
	**FC**	**Adj. *P***	**FC**	**Adj. *P***	**FC**	**Adj. *P***	**FC**	**Adj. *P***
**Air**								
SAA1	1.5	2.5×10^{-4}	1.6	4.1×10^{-3}	2.0	6.0×10^{-1}	1.5	1.0×10^{0}
SAA2	1.1	1.9×10^{-2}	1.5	1.7×10^{-10}	1.9	1.0×10^{0}	1.2	1.0×10^{0}
S100A9	0.8	1.0×10^{0}	1.4	1.0×10^{-3}	1.8	1.3×10^{-12}	1.0	1.0×10^{0}
LCN2	0.7	1.9×10^{-1}	1.3	1.1×10^{-11}	1.3	4.5×10^{-1}	0.9	1.0×10^{0}
CXCL6	1.0	1.0×10^{0}	1.1	5.9×10^{-3}	1.3	1.0×10^{0}	1.1	1.0×10^{0}
CCL20	1.2	1.0×10^{0}	-	-	1.1	1.0×10^{0}	-	-
CXCL1	1.1	1.0×10^{0}	1.2	3.4×10^{-6}	1.1	1.0×10^{0}	1.2	1.0×10^{0}
**Smoke**								
SAA1	2.4	7.2×10^{-24}	1.2	3.9×10^{-83}	1.5	2.5×10^{-43}	1.7	2.8×10^{-59}
SAA2	1.7	7.5×10^{-16}	1.2	5.8×10^{-74}	1.5	9.5×10^{-17}	1.6	5.9×10^{-40}
S100A9	1.1	1.0×10^{0}	1.5	7.9×10^{-13}	1.4	1.0×10^{0}	1.2	3.0×10^{-16}
LCN2	1.3	1.7×10^{-5}	1.7	5.5×10^{-11}	1.3	2.0×10^{-38}	1.2	1.7×10^{-16}
CXCL6	1.5	5.1×10^{-14}	1.4	1.0×10^{0}	2.4	2.2×10^{-12}	1.8	1.0×10^{0}
CCL20	1.6	5.5×10^{-4}	1.4	4.3×10^{-3}	1.2	4.2×10^{-32}	1.1	5.7×10^{-24}
CXCL1	1.7	1.3×10^{-9}	1.5	9.4×10^{-1}	1.3	6.9×10^{-5}	1.1	5.7×10^{-15}

Differential gene expression results of the pro-inflammatory gene set that was differentially expressed between COPD and HC donors of the acute smoke model in air control and smoke-exposed ALI cultures of the chronic smoke model.

Table S2. Fraction of air control and smoke-exposed cells expressing SARS-CoV-2 infection associated genes.

Model	Genes	Cell type	P_{Smoke}	P_{Group}	$P_{Group:Smoke}$	Air (%)	Smoke (%)
**Acute**	***ACE2***	SC	$1.8x10^{-1}$	$9.0x10^{-1}$	$8.9x10^{-1}$	1.2	2.0
		CC	$2.0x10^{-3}$	$6.0x10^{-1}$	$4.4x10^{-1}$	1.2	2.2
		BC	$5.3x10^{-1}$	$7.5x10^{-1}$	$6.8x10^{-1}$	1.5	2.0
		IC	$4.1x10^{-2}$	$1.0x10^{0}$	$4.4x10^{-1}$	1.0	1.9
	BSG	SC	$2.1x10^{-3}$	$8.5x10^{-1}$	$6.5x10^{-1}$	69.6	77.1
		CC	$5.8x10^{-3}$	$3.7x10^{-1}$	$2.5x10^{-1}$	74.4	79.1
		BC	$1.9x10^{-3}$	$9.2x10^{-1}$	$2.9x10^{-1}$	89.1	95.5
		IC	$1.1x10^{-2}$	$6.8x10^{-1}$	$6.6x10^{-1}$	84.5	91.4
	CTSL	SC	$7.8x10^{-2}$	$5.5x10^{-1}$	$5.6x10^{-1}$	9.9	11.0
		CC	$8.3x10^{-1}$	$1.0x10^{0}$	$8.6x10^{-2}$	26.7	28.2
		BC	$8.5x10^{-1}$	$7.5x10^{-1}$	$6.9x10^{-1}$	18.3	20.5
		IC	$8.3x10^{-1}$	$8.4x10^{-1}$	$3.5x10^{-1}$	14.8	16.2
	TMPRSS2	SC	$2.2x10^{-1}$	$5.7x10^{-1}$	$7.8x10^{-1}$	22.6	23.6
		CC	$2.1x10^{-2}$	$6.3x10^{-1}$	$1.0x10^{0}$	37.8	42.4
		BC	$2.6x10^{-1}$	$5.5x10^{-1}$	$7.9x10^{-1}$	9.2	10.5
		IC	$4.4x10^{-1}$	$7.0x10^{-1}$	$7.9x10^{-1}$	18.3	20.5
	TMPRSS4	SC	$3.5x10^{-4}$	$7.8x10^{-1}$	$3.5x10^{-1}$	30.8	46.8
		CC	$1.1x10^{-3}$	$3.3x10^{-1}$	$3.7x10^{-1}$	33.1	42.6
		BC	$9.8x10^{-4}$	$7.6x10^{-1}$	$1.0x10^{0}$	44.6	64.9
		IC	$1.1x10^{-3}$	$8.4x10^{-1}$	$5.9x10^{-1}$	48.0	68.4

Model	Genes	Cell type	P_{Smoke}	P_{Group}	$P_{Group:Smoke}$	Air (%)	Smoke (%)
**Chronic**	***ACE2***	SC	8.8×10^{-1}	6.7×10^{-1}	8.9×10^{-1}	2.1	2.2
		CC	2.9×10^{-3}	9.2×10^{-1}	1.0×10^{0}	1.6	4.0
		BC	1.5×10^{-2}	4.4×10^{-1}	6.1×10^{-1}	2.2	3.7
		IC	8.1×10^{-2}	5.3×10^{-1}	3.9×10^{-1}	2.1	3.2
	BSG	SC	2.7×10^{-2}	6.5×10^{-1}	6.4×10^{-1}	64.6	82.9
		CC	9.7×10^{-2}	5.4×10^{-1}	2.9×10^{-1}	75.8	81.9
		BC	2.5×10^{-1}	7.0×10^{-1}	5.3×10^{-1}	90.7	96.3
		IC	2.0×10^{-1}	8.4×10^{-1}	8.3×10^{-1}	79.8	85.9
	CTSL	SC	5.4×10^{-3}	7.6×10^{-1}	8.5×10^{-1}	10.2	20.9
		CC	9.4×10^{-2}	6.7×10^{-1}	5.2×10^{-1}	28.5	32.9
		BC	2.2×10^{-4}	9.2×10^{-1}	1.0×10^{0}	22.8	38.5
		IC	2.6×10^{-1}	4.3×10^{-1}	8.8×10^{-1}	13.3	21.0
	TMPRSS2	SC	5.4×10^{-4}	1.0×10^{0}	1.0×10^{0}	23.0	37.8
		CC	3.2×10^{-2}	3.6×10^{-1}	1.0×10^{0}	36.5	45.1
		BC	8.9×10^{-1}	8.9×10^{-1}	6.1×10^{-1}	8.5	9.1
		IC	1.4×10^{-2}	8.5×10^{-1}	3.5×10^{-1}	18.7	27.9
	TMPRSS4	SC	1.3×10^{-3}	6.6×10^{-1}	5.0×10^{-1}	32.4	60.9
		CC	8.9×10^{-5}	5.0×10^{-1}	3.0×10^{-1}	33.8	55.8
		BC	9.5×10^{-4}	3.0×10^{-1}	4.2×10^{-1}	51.0	75.1
		IC	8.6×10^{-3}	6.4×10^{-1}	8.5×10^{-1}	58.2	76.5

Summary of statistical analysis investigating the impact of donor group (Group; COPD/HC) and smoke exposure (Smoke; Air/Smoke) on the fraction of secretory (SC), ciliated (CC), basal (BC) and intermediate (IC) cells that express SARS-CoV-2 infection associated genes. Data are shown for the acute and chronic smoke model. Percentage of air control (% Air) and smoke-exposed (Smoke %) cells expressing the respective gene reflects the average percentage across all donors (COPD and HC donors).

Table S3. Characteristics of the SAEC basal cell donors.

Donor ID	Disease status	Age (years)	Sex	Race	Smoking
**A**	Healthy control	67	Female	Caucasian	No
**B**	Healthy control	42	Female	Hispanic	No
**C**	Healthy control	38	Male	Caucasian	No
**D**	COPD	57	Female	Caucasian	Yes
**E**	COPD	51	Female	Caucasian	Yes
**F**	COPD	62	Female	Black	Yes
